# Supplementary figures and images for: Applying 3D correlative structured illumination microscopy and X-ray tomography to characterise herpes simplex virus-1 morphogenesis
Source: eLife. 2025 Dec 19;14:RP105209. doi: 10.7554/eLife.105209 (PMC12716837; doi:10.7554/eLife.105209)

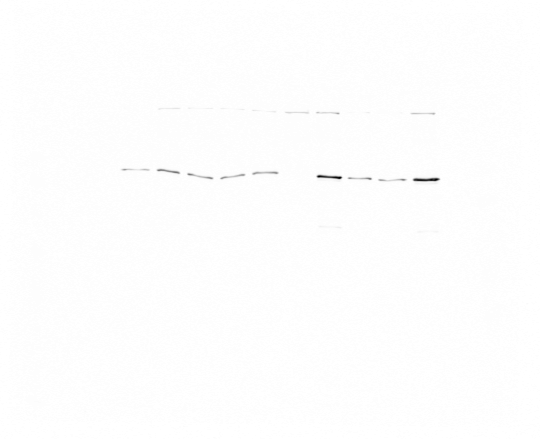

Supplement: Figure 1—source data 1. [file elife-105209-fig1-data1.zip › Figure 1-source data 1_8 bit/VP16_Fig1C.tif]

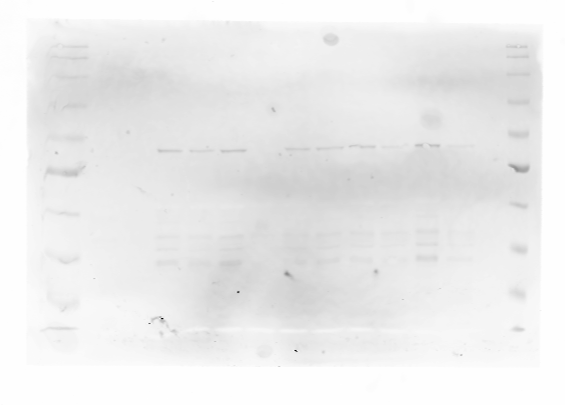

Supplement: Figure 1—source data 1. [file elife-105209-fig1-data1.zip › Figure 1-source data 1_8 bit/UL21_Fig1C.tif]

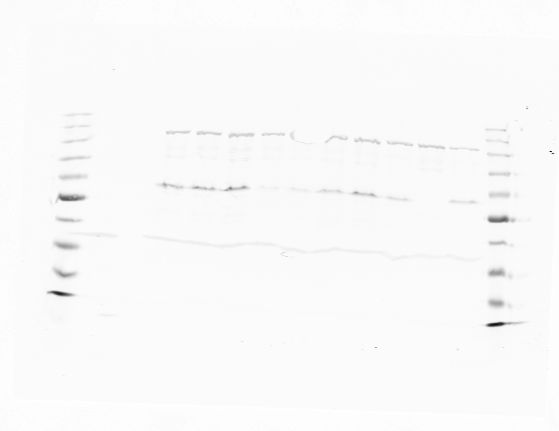

Supplement: Figure 1—source data 1. [file elife-105209-fig1-data1.zip › Figure 1-source data 1_8 bit/US3_GAPDH_VP5_Fig1C.tif]

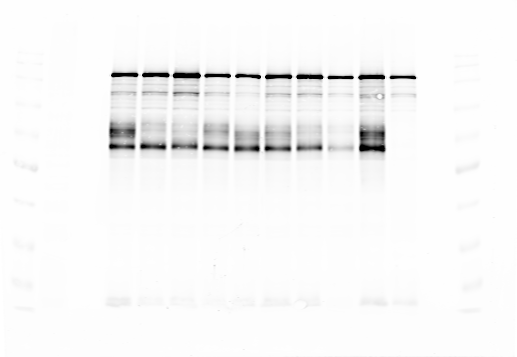

Supplement: Figure 1—source data 1. [file elife-105209-fig1-data1.zip › Figure 1-source data 1_8 bit/VP5_Fig1C.tif]

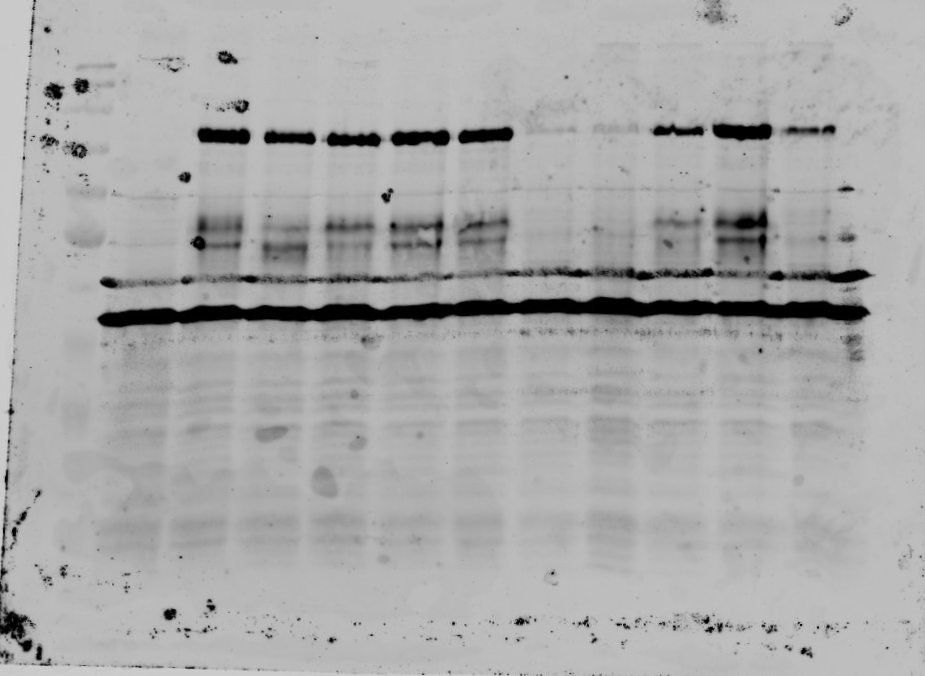

Supplement: Figure 1—source data 1. [file elife-105209-fig1-data1.zip › Figure 1-source data 1_8 bit/GAPDH_Fig1D.tif]

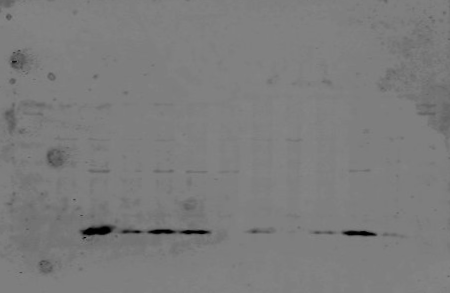

Supplement: Figure 1—source data 1. [file elife-105209-fig1-data1.zip › Figure 1-source data 1_8 bit/UL34_Fig1D.tif]

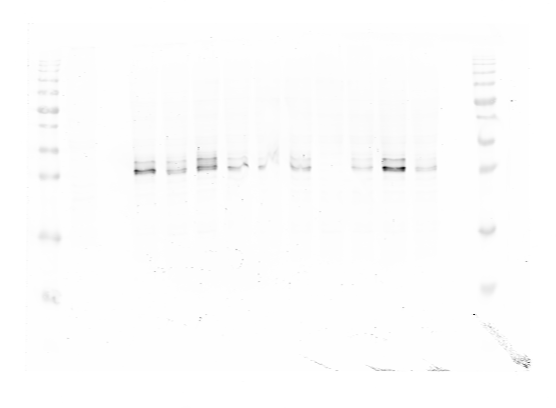

Supplement: Figure 1—source data 1. [file elife-105209-fig1-data1.zip › Figure 1-source data 1_8 bit/UL51_Fig1C.tif]

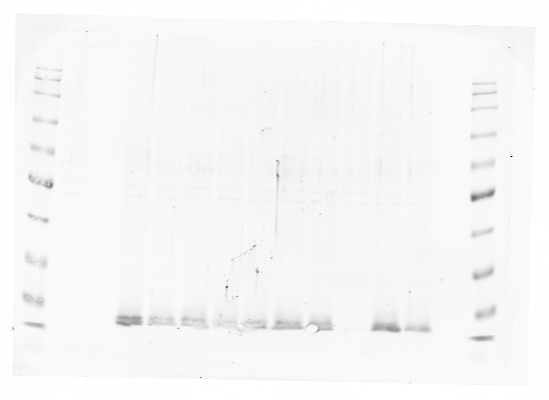

Supplement: Figure 1—source data 1. [file elife-105209-fig1-data1.zip › Figure 1-source data 1_8 bit/UL20_Fig1C.tif]

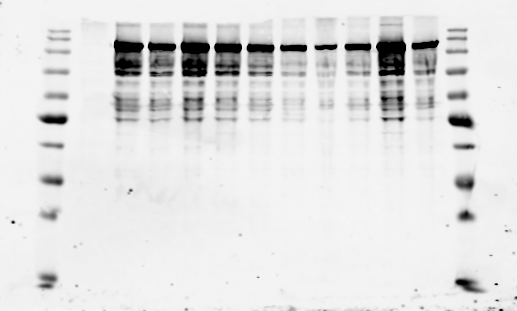

Supplement: Figure 1—source data 1. [file elife-105209-fig1-data1.zip › Figure 1-source data 1_8 bit/VP5_Fig1D.tif]

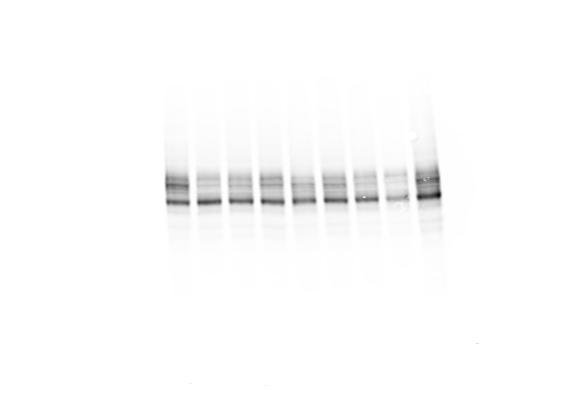

Supplement: Figure 1—source data 1. [file elife-105209-fig1-data1.zip › Figure 1-source data 1_8 bit/gE_Fig1C.tif]

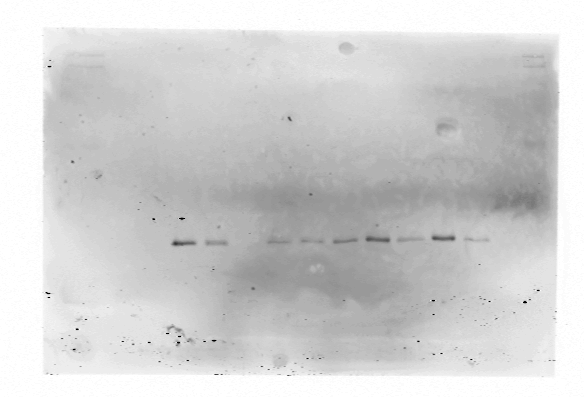

Supplement: Figure 1—source data 1. [file elife-105209-fig1-data1.zip › Figure 1-source data 1_8 bit/UL16_Fig1C.tif]

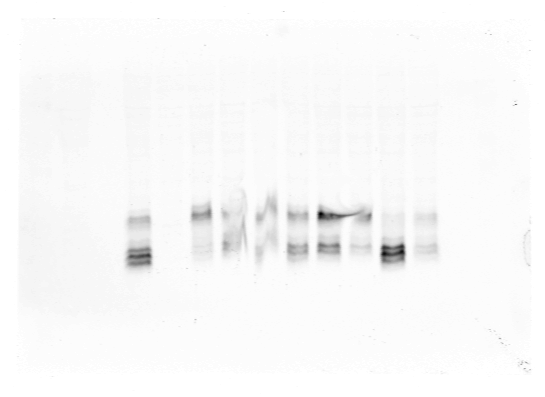

Supplement: Figure 1—source data 1. [file elife-105209-fig1-data1.zip › Figure 1-source data 1_8 bit/UL11_Fig1C.tif]

## Slide 1
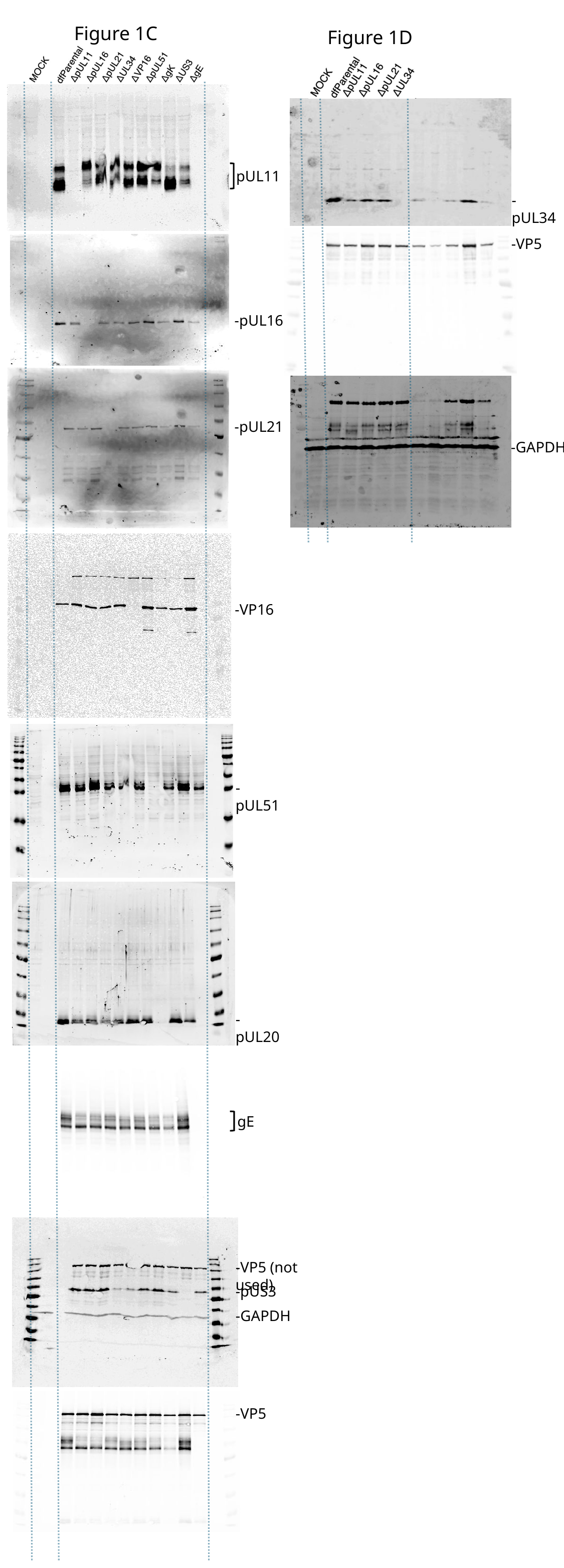

Figure 1C
Figure 1D
pUL11
-pUL34
-VP5
-pUL16
-pUL21
-GAPDH
-VP16
-pUL51
-pUL20
gE
-VP5 (not used)
-pUS3
-GAPDH
-VP5

Supplement: Figure 1—source data 2. [file elife-105209-fig1-data2.zip › Figure 1-source data 2/Figure 1-source data 2.pptx]
