# Supplementary material for: Applying 3D correlative structured illumination microscopy and X-ray tomography to characterise herpes simplex virus-1 morphogenesis
Source: eLife. 2025 Dec 19;14:RP105209. doi: 10.7554/eLife.105209 (PMC12716837; doi:10.7554/eLife.105209)
Supplement: Supplementary file 1. — (A) Primers to generate mutants by two-step red recombination. Sequences homologous to the gene are in upper case, sequences homologous to the template plasmids pEP-KanS or pEP-RenillaLuc are in lower case, mutated start codons are underlined, and introduced stop codons are in bold. (B) PCR amplification primers for HSV-1 US3 and UL53. (C) Sanger sequencing primers for HSV-1 US3 and UL53. [file elife-105209-supp1.docx]

**Supplementary file 1A. Primers to generate mutants by two-step red recombination.** Sequences homologous to the gene are in upper case, sequences homologous to the template plasmids pEP-KanS or pEP-RenillaLuc are in lower case, mutated start codons are underlined and introduced stop codons are in bold.

| **Gene** | **Forward primer (5′ to 3′)** | **Reverse primer (5′ to 3′)** |
| --- | --- | --- |
| UL11 | GAAGCAGCCGCCTGGCGTTCGACGACACGCTCGCCGAGCTctgggc**tag**TCGTTCTCCGGGACCCGGCCaggatgacgacgataagtaggg | CGTTGTTTCGGCAGCAGCAGGGCCGGGTCCCGGAGAACGA**cta**gcccagAGCTCGGCGAGCGTGTCGTCcaaccaattaaccaattctgattag |
| UL16 | GGCGCAGCTGGGACCCCGGCGGCCCCTGGCGCCGCCTGGT**TGATAGTAA**GCTTGCCCCGGCCGGATTCCCaggatgacgacgataagtaggg | CGTGCCGCGAGCTCCGGCCCGGGAATCCGGCCGGGGCAAGC**TTACTATCA**ACCAGGCGGCGCCAGGGGCCcaaccaattaaccaattctgattag |
| UL21 | GCACTACCGGGACGTTGTGTTTTACGTCACAACGGACCGA**TGATAGTAA**GCTTTGTGTGCGGGGGGTGTGaggatgacgacgataagtaggg | CGGCCGCCCCACGGAATAAACACACCCCCCGCACACAAAGC**TTACTATCA**TCGGTCCGTTGTGACGTAAAcaaccaattaaccaattctgattag |
| UL34 | CCCTTTGGTGGGTTTACGCGGGCACGCACGCTCCCATCGCGGGCGCCatgacttcgaaagtttatgatcc | GCTTAAGACCCCGCAGGGCCTGGTGCCACGGGCGGGAGGGCCCTTGGGTTTTAttgttcatttttgagaactcgc |
| UL48 | CAAAAGCCCGATATCGTCTTTCCCGTATCAACCCCACCCAGAATTCTTTACCGATGCCCTTGGAATaggatgacgacgataagtaggg | CCTACCCACCGTACTCGTCAATTCCAAGGGCATCGGTAAAGAATTCTGGGTGGGGTTGATACGGGAcaaccaattaaccaattctgattag |
| UL51 | TATATGTGGCTGGGGAGCGCGCCCCGAGGAACAATATGAG**TAGTGATAA**GGATCCGTTCCGCCCTCGGAGGCGGAaggatgacgacgataagtaggg | GGGCCTCCTGCAGCCGCGGCTCCGCCTCCGAGGGCGGAACGGATCC**TTATCACTA**CTCATATTGTTCCTCGGGGCcaaccaattaaccaattctgattag |
| UL53 | GGTACGCCCCACCGGCACCAACAACGACACCGCCCTCGTG**TGATAGTAA**GCTTACCAGACCCTATTGTTTCTGaggatgacgacgataagtaggg | GGGGGGTGCGTCGGGGCCCCCAGAAACAATAGGGTCTGGTAAGC**TTACTATCA**CACGAGGGCGGTGTCGTTGTcaaccaattaaccaattctgattag |
| US3 | CACCACACCACCCGGCGATGCCGAGCGCCTGTGTCATCTGTGATCTTCGAGACTGCCGTCaggatgacgacgataagtaggg | GAGAACAAGGACGCGTTGTGGACGGCAGTCTCGAAGATCACAGATGACACAGGCGCTCGGcaaccaattaaccaattctgattag |
| US8 | GGGGTTTCTTCTCGGTGTTTGTGTTGTATCGTGCTTGGCG**TAGTGATAA**GCTTCGTCCTGGAGACGGGTGAGTaggatgacgacgataagtaggg | AACGAAACGTCCTCGCCGACACTCACCCGTCTCCAGGACGAAGC**TTATCACTA**CGCCAAGCACGATACAACACcaaccaattaaccaattctgattag |

**Supplementary file 1B. PCR amplification primers for HSV-1 US3 and UL53.**

| **Gene** | **Forward primer (5′ to 3′)** | **Reverse primer (5′ to 3′)** |
| --- | --- | --- |
| UL53 | GGTCCTCCTACAGCTAGTCC | GCTGGGTTGGTCTTGGTAAC |
| US3 | CAGATTTGTAAGGCCACGCAC | GATAATGGGAACAACGGCACG |

**Supplementary file 1C. Sanger sequencing primers for HSV-1 US3 and UL53.**

| **Gene** | **Complementary strand** | **Primer (5′ to 3′)** |
| --- | --- | --- |
| UL53 | Sense | CAAATGCGACAGCAACCG |
|  | Sense | CTCTTGAACTACGCAGGC |
| US3 | Sense | CTGCCGCTCCTTAAAACC |
|  | Sense | CAGAAGAGCTGGACGCCATG |
|  | Sense | GATCAAGCCCCTTCCCCTAC |
|  | Sense | CCACCGCGACATTAAGAC |
|  | Anti-sense | GTGATCTGACTGTCGCACG |
